# Oral mucosa and Streptococcus mutans count in the saliva. Does graphene oxide nanoparticle mouthwash have a good effect?

**DOI:** 10.22088/cjim.12.3.342

**Published:** 2021-04

**Authors:** Fatemeh Eshaghi Gorji, Maryam Seyedmajidi, Fariba Asgharpours, Hamed Tashakorian, Ali-akbar Moghadamnia, Sohrab Kazemi, Homayoon Alaghehmand

**Affiliations:** 1 Dental Materials Research Center, Health Research Institute, Babol University of Medical Sciences, Babol, Iran; 2 Department of Laboratory Sciences, Faculty of Para-medicine, Babol University of Medical Sciences, Babol, Iran; 3 Cellular and Molecular Biology Research Center (CMBRC), Health Research Institute, Babol University of Medical Sciences, Babol, Iran; 4 Department of Pharmacology, School of Medicine, Babol University of Medical Sciences, Babol, Iran; 5 Neuroscience Research Center, Health Research Institute, Babol University of Medical Sciences, Babol, Iran

**Keywords:** Nanoparticles, Streptococcus mutans, Sodium fluoride

## Abstract

**Background:**

This study aimed to assess the effect of graphene oxide (GO) nanoparticles mouthwash on oral mucosa, Streptococcus mutans (S. mutans) count in the saliva of rats, and human enamel surface microhardness, in comparison with fluoride mouthwash.

**Methods:**

This study was conducted in two phases namely an animal study, and an in vitro experimental study. GO mouthwash (0.005%), sodium fluoride (NaF) mouthwash (0.05%), and a combination of both (0.05% NaF-0.005% GO) were prepared. The oral cavity of 36 rats was inoculated with S. mutans, and they were randomly divided into 4 groups according to the type of mouthwash. The control group received saline mouthwash. Fourteen days after using the mouthwashes, all rats were sacrificed, and the salivary S. mutans count was measured. The buccal and tongue mucosa were also histologically examined for the type and severity of inflammation, number of blood vessels, epithelial thickness, and epithelial keratinization. For microhardness testing, 40 sound extracted human premolars were randomly assigned to four groups (n=10) of culture medium with S. mutans and different mouthwashes. The enamel microhardness was measured at 7 and 14 days, and compared with the baseline value.

**Results:**

The mean S. mutans count in the saliva of rats in GO and NaF-GO groups was significantly lower than that in other groups (p<0.001). Enamel microhardness in NaF and NaF-GO groups significantly increased at 7 and 14 days, compared with baseline.

**Conclusion:**

Addition of GO nanoparticles improved the antibacterial properties without causing adverse mucosal effects such as ulceration, acute inflammation or atrophy of the epithelium of the oral mucosa, but had no effect on surface hardness of the enamel.

Development of dental caries and periodontal disease is closely associated with the activity of pathogenic microorganisms (1). Streptococcus mutans (S. mutans) is a Gram-positive, facultative anaerobe, and a major culprit responsible for the development of dental caries. It produces high amounts of organic acids that decrease the pH of the oral cavity (2). Thus, attempts are ongoing to find antimicrobial agents with bactericidal or bacteriostatic properties against S. mutans (3). Graphene was introduced as an antibacterial agent with strong antibacterial activity against many bacterial species (2, 4). Graphene nanoparticles (GNPs) are carbon allotropes synthesized from graphene sheets two-dimensionally with a thickness of 2-10 nm and dimensions of 1-10 m (5).

The antimicrobial properties of GNPs against both Gram-negative (Pseudomonas aeruginosa) and Gram-positive (S. mutans) bacteria have been previously documented (5, 6). Insignificant cytotoxicity of GNPs has also been documented in vivo by using the Caenorhabditis elegans model system (5). Regarding the cytotoxicity of different concentrations of graphene oxide (GO), evidence shows that 50 µg/mL concentration may be the toxicity threshold of GO for mammal cells. Concentrations over 50 µg/mL may damage the human fibroblasts and T-lymphocytes (7-9). 

Graphene is potentially toxic due to its super hydrophobicity. However, its toxicity can be decreased by functionalization, which also improves its water solubility (10). Considering the significance of this topic, this study aimed to assess the antimicrobial properties of GNPs against S. mutans and also analyze the effects of GNPs on the oral mucosa of rats, and surface microhardness of human enamel, in comparison with sodium fluoride (NaF) mouthwash. 

## Methods

This study was conducted in two phases namely an animal study, and an in vitro, experimental study. The study was conducted in accordance with the Declaration of Helsinki and the guidelines for the care and use of laboratory animals. The study was approved by the Ethics Committee of Babol University of Medical Sciences (IR.MUBABOL.HRI.REC.1398.309). 

**Figure 1:**
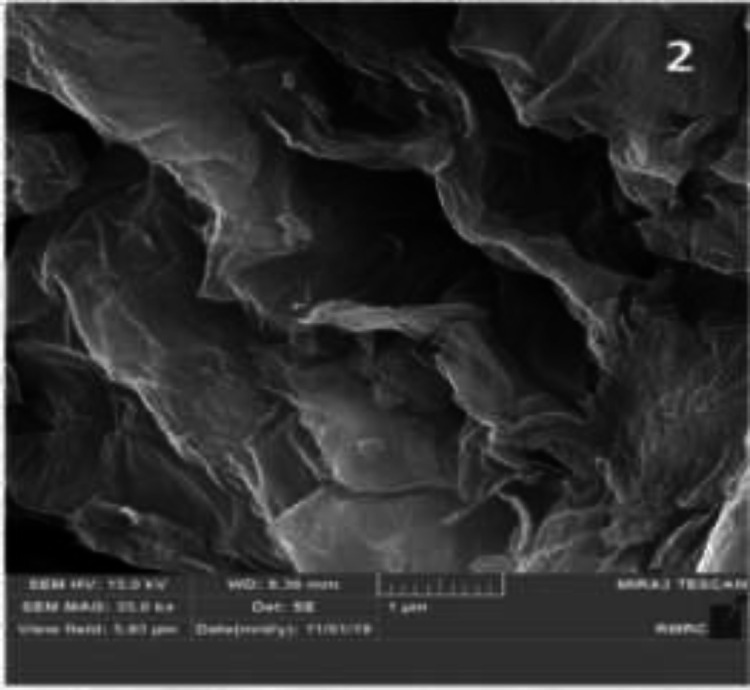
SEM micrograph of GNPs at x35k magnification

**Figure 2:**
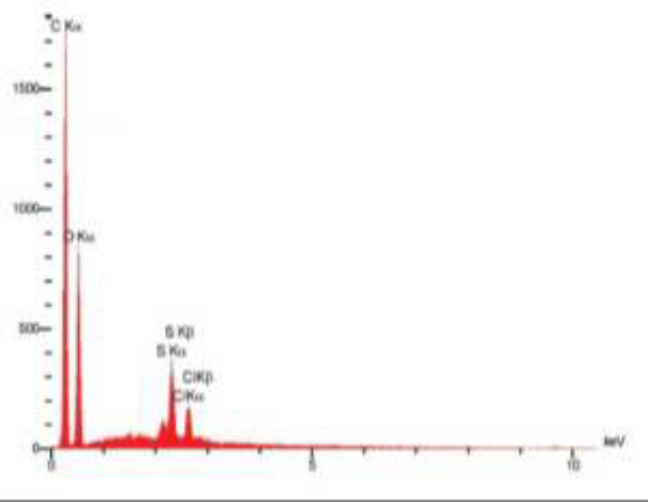
XRD of GNPs: The X axis shows the intensity (a.u.) and the Y axis shows energy (eV). Carbon is dominantly seen in this graph

**Characterization of GO:** GO powder with 3.4-7 nm particle size and layered structure was obtained from US-nano company (USA). Scanning electron microscopic imaging (JSM 6701F, JEOL) was performed to assess the morphology of GNPs (Figure 1). X-ray photoelectron spectroscopy (PHI-5702; XPS, Physical Electronics) by A1-Ka as the x-ray radiation source and Au binding energy reference (Au 4f7 / 2: 84.00 eV) was also performed to assess the purity percentage of materials (figure 2). 

**Preparation of GO and NaF mouthwashes and a combination of both (NaF-GO):** To functionalize (silanize) the nano-GO, 0.5 g of nano-GO (graphene powder with a particle size of 3.4-7 nm with layered structure obtained from US-nano company) was dispersed in 25 mL of toluene solvent. To separate the GO sheets from each other and increasing the efficiency of functionalization, the mixture was ultrasonicated for 5 min. Next, 0.75 mL of 3-mercaptopropyl trimethoxysilane was added to the mixture and stirred for 30 h under reflux conditions. It was then centrifuged at 5000 rpm for 15 min. The supernatant was discarded, and the functionalized nano-GO deposit was rinsed with acetone solvent twice. The obtained compound was desiccated in an oven at 60°C for 24 h. To prepare 0.005% GO mouthwash, 5 mg of functionalized nano-GO in 100 ml of distilled water was used. To prepare 0.05% NaF, and 0.05% NaF-0.005% GO mouthwashes, 2 mg of NaF in 10 mL of distilled water was used. 

**Animal (in vivo) study: **The animal study was conducted on 36 adult male Wistar rats. The following formula was used for sample size calculation: 

n = z (1-α)^2^ p (1 – p) / d^2^

z (1-α)^2^ =3.84, p=0.05, 1-p=0.95, d=0.06

 The rats were systemically healthy, aged 8-12 weeks, and weighed 150-200 g. They were kept under standard environmental conditions (22±2°C temperature, 55±5% humidity, and 12-h light/12-h dark cycles) with ad libitum access to standard food and water (Nuvilab CR-1, PR, Brazil). Also, the rats were caged individually during the experiment. 

At the onset of the study, the rats underwent hard and soft tissue examination. Also, they received a triple drug regimen (1 g/kg ampicillin, chloramphenicol, and carbenicillin) for 3 consecutive days to become germ-free and eliminate S. mutans from their oral cavity (11). The oral cavity of the rats was then inoculated with S. mutans (ATCC 35668) suspension (obtained from the Iranian Research Organization for Science and Technology) once a day for 3 consecutive days, using a swab. During the process of inoculation, they received a nutritional regimen containing 40% sucrose (Asia Pajouhesh, Iran) (12, 13). Saliva samples were collected from the rats 24 h after their inoculation. The samples were dissolved in brain heart infusion (BHI) broth. To ensure inoculation of S. mutans, the samples were cultured on Mitis Salivarius-mutans valinomycin agar, and the presence of S. mutans on the culture medium was confirmed using Microgen STREP-ID kits (Microgen bioproducts ,UK) (14). The rats were then randomly divided into 4 groups as follows: Group 1 received saline mouthwash (control group); group 2 received 0.05% alcohol-free NaF mouthwash; group 3 received 0.005% alcohol-free GO mouthwash, and group 4 received a combination of NaF-GO mouthwash. 

Mouthwashes were applied by a sterile swab once a day for 14 days. Also, 10% sucrose was added to the drinking water of rats to enhance the growth and proliferation of S. mutans. After 14 days, the rats were anesthetized by intraperitoneal injection of 10% ketamine (40 mg/kg; Alfasan, Woerden, Holland) and 2% xylazine (5 mg/kg; Alfasan, Woerden, Holland). Saliva samples were then collected from the rats by a sterile swab (3 times), dissolved in BHI broth, and after dilution of the primary culture, the number of S. mutans colonies was counted. 

After anesthesia induction with high-dose chloroform, the rats were sacrificed and beheaded. After immersion in 10% formalin for 1 week for tissue fixation, they were sent to a pathology laboratory for histological analysis. Any visible mucosal change was recorded. Also, the buccal and tongue mucosa were histologically analyzed for the type and severity of inflammation, number of blood vessels, epithelial thickness, and epithelial keratinization. 

**In vitro, experimental phase of the study (assessment of the effect of GO mouthwash on enamel microhardness):**

Based on previous studies(15), 40 sound human premolars extracted due to orthodontic treatment or periodontal disease with no sign of caries, white spot lesions, or brown spots were used for this study. S. mutans (ATCC 35668) obtained from the Iranian Research Organization for Science and Technology was cultured in BHI broth at 37°C under anaerobic conditions (10% CO2, 10% H2, 80% N2). To create S. mutans biofilm, 1% sucrose (Asia Pajouh, Iran) was added to BHI broth (1% BHIS). 

Prior to the experiment, the teeth were immersed in 75% alcohol for 12 h and were then sterilized by UV radiation for 2 h. The teeth were then mounted in epoxy resin, and their occlusal surface was reduced to the central groove in buccolingual direction. Next, the surface of the teeth was polished with up to 4000-grit silicon carbide abrasive papers. The entire tooth surface, except for the occlusal surface, was coated with nail varnish. 

To form salivary pellicle, all teeth were stored in sterile customized artificial saliva (containing 2 mg/L C6H8O6, 30 mg/L C6H12O6, 580 mg/L NaCl, 170 mg/L CaCl2, 1270 mg/L KCl, 160 mg/L NaSCN, 330 mg/L KH2PO4, 200 mg/L CH4N2O, 340 mg/L Na2HPO4, amd 1000 mL deionized water (16) at 37°C for 2 h. 

Prior to the experiment, enamel microhardness was measured at three points below the hypothetical buccal cusp tip in a microhardness tester (Koopa, Iran) by applying 500 g force for 10 s. The mean of the three values was calculated and served as the baseline microhardness value. The diagonal length of each indentation was directly measured by using an optical lens. The Vickers microhardness number (kgf/mm^2^) was calculated using the equation below (17):

VHN=1854.4Pd2 (17)

Where P is the force (g), and d is the diagonal length (m). 

Next, the teeth were randomly divided into four groups (n=10) for immersion in the following solutions (containing 10 mL of each mouthwash, 1 mL of artificial saliva, and 1 mL of 10^6^ CFUs/mL S. mutans grown in 1% BHIS suspension) as follows: group 1 was inoculated with S. mutans grown in 1% BHIS and artificial saliva as negative control. 

Groups 2-4 were inoculated with S. mutans grown in 1% BHIS, in the presence of different mouthwash namely 0.05% NAF, 0.005% G, and a combination of 0.05%NAF-0.005% G and artificial saliva. The teeth were then incubated at 37°C for 14 days in the abovementioned condition. The solutions were refreshed once a day. The microhardness of the teeth was measured after 7 and 14 days as explained earlier. 

**Statistical analysis: **Data were analyzed using SPSS (SPSS Inc., IL, USA) by repeated measures ANOVA, Bonferroni test, ANOVA, Tukey’s post-hoc test, Chi-square test, and Fisher’s exact test. A p<0.05 was considered statistically significant.

## Results

**Characterization of nanoparticles:** Figure 1 shows the thin sheets of nano-GO with 4-7 nm thickness, large length and 10-50 µm width. Figure 2 shows the x-ray diffraction (XRD) analysis of the powder used in this study. Table 1 shows the results of energy dispersive x-ray spectroscopy (EDS). 

**Table 1:** EDS results indicating the weight percentage of elements in GO powder

**Element**	**GO powder**
C	60.12
O	36.04
S	2.43
Cl	1.41
Zn	-

**In vivo study results:** The mean S. mutans count (CFUs/mL) was significantly different in different mouthwash groups p<0.001, table 2). The mean S. mutans count in the saliva of rats in 0.005% GO (P<0.001) and 0.005% GO-0.05% NaF (P<0.001) groups was significantly lower than that in the control group. The mean S. mutans count was not significantly different in 0.05% NaF and control groups (P=0.689). The mean S. mutans count in 0.005% GO mouthwash group was not significantly different from that in 0.005% GO-0.05% NaF mouthwash group (P=0.988). 

**Table 2:** Mean count of S. mutans in the study groups (n=9)

**Groups**	**Mean±std. deviation**	**Minimum**	**Maximum**
Control	4472.22±1959.610	2300	7800
0.05% NaF	3733.33±2042.364	2000	8000
0.005% GO	1022.22±167.912	800	1250
0.05% NaF +0.005% GO	805.56±101.379	650	1000

P value<0.001

**In vitro study results (enamel microhardness): **According to repeated measures ANOVA, the enamel microhardness was significantly different at baseline and 7 and 14 days in the control group (p<0.001, table 3). According to the Bonferroni test, the enamel microhardness in the control group significantly decreased at 14 days, compared with baseline and 7 days (p<0.001), but the mean microhardness was not significantly different at baseline and 7 days (P=0.098). According to repeated measures ANOVA, the enamel microhardness was significantly different at baseline and 7 and 14 days in 0.05% NaF group (p<0.001). According to the Bonferroni test, the enamel microhardness in 0.05% NaF group significantly increased at 14 days compared with baseline (P<0.001). Also, the mean microhardness at 14 days was significantly higher than that in 7 days (P=0.008). According to repeated measures ANOVA, the enamel microhardness was not significantly different at baseline and 7 and 14 days in 0.005% GO group (P=0.237). According to repeated measures ANOVA, the enamel microhardness was significantly different at baseline and 7 and 14 days in 0.05% NaF-0.005% GO group (p<0.001). According to the Bonferroni test, the enamel microhardness in this group significantly increased in 14 days and also in 7 days compared with baseline (p<0.001). Also, the mean microhardness in 14 days was significantly higher than that in 7 days (p<0.001). 

**Table 3:** Mean Vicker’s enamel microhardness in the study groups (n=10) at different time points, (kgf/mm2)

**Groups**	**Baseline**	**7 days**	**14 days**	P value*
Control	285.830±34.409	276.377±35.783	172.232±34.921	<0.001
0.05% NaF	281.897±28.639	287.531±30.515	332.964±41.202	<0.001
0.005% GO	306.633±38.907	310.031±41.274	312.297±37.561	0/237
0.05% NaF + 0.005% GO	276.864±47.457	317.565±38.770	362.998±38.013	<0.001
P value**	0/329	0/059	<0.001	

*Repeated measures ANOVA; *ANOVA

According to ANOVA, enamel microhardness of the groups was not significantly different at baseline (P=0.329) or in 7 days (P=0.059). However, the difference in this respect was significant at 14 days (p<0.001, table 3). The Tukey’s test revealed that the mean enamel microhardness in 0.05% NaF (p<0.001), 0.005% GO (p<0.001), and 0.005% GO-0.05% NaF (p<0.001) groups was significantly higher than that in the control group. 

The mean enamel microhardness was not significantly different in 0.05% NaF and 0.005% GO-0.05% NaF groups (P=0.305). The mean enamel microhardness in 0.005% GO group was significantly lower than that in 0.005% GO-0.05% NaF group (P=0.025). Effects of 0.005% GO, 0.05% NaF and NaF-GO mouthwashes on the oral mucosa of rats: No ulceration was noted in any rat (100%). Table 4 shows the pathological findings.

**Table 4:** Pathological analysis of the oral mucosa of the rats

		**C (n=18)**	**NaF (n=18)**	**GO (n=18)**	**NaF-GO (n=18)**	**P value**
Type of inflammation	No inflammation	17 (94.4%)	13 (72.2%)	3 (16.7%)	1 (5.6%)	<0.001*
	Chronic	1 (5.55%)	5 (27.8%)	15 (83.3%)	17 (94.4%)	
Severity of inflammation	No inflammation	17 (94.4%)	13 (72.2%)	3 (16.7%)	1 (5.6%)	<0.001**
	Mild	1 (5.55%)	5 (27.8%)	12 (66.7%)	15 (83.3%)	
	Moderate	0 (0.0%)	0 (0.0%)	3 (16.7%)	2 (11.1%)	
Epithelial thickness	Thin	17 (94/4%)	10 (55.6%)	9 (50.0%)	8 (44.4%)	0.668**
	Moderate	1 (5.55%)	8 (44.4%)	8 (44.4%)	8 (44.4%)	
	Thick	0 (0.0%)	0 (0.0%)	1 (5.6%)	2 (11.1%)	
Keratinization	Absent	17 (94.4%)	13 (72.2%)	4 (22.2%)	1 (5.6%)	<0.001*
	Present	1 (5.55%)	5 (27.8%)	14 (77.8%)	17 (94.4%)	

*: Chi-square test, **: Fisher's exact test

## Discussion

**In vivo test results:** The results of the present study indicated that application of 0.05% NaF mouthwash insignificantly decreased the S. mutans count in the saliva of rats, compared with the control group. Fluoride was introduced to dentistry since it can effectively decrease demineralization and increase remineralization of tooth structure. Fluoride inhibits the glycolytic enzyme that converts 2-P-glycerate to phosphoenolpyruvate, and interferes with the metabolism and proliferation of bacteria as such. 

Also, fluoride ions inhibit the synthesis of glycosyltransferase enzyme. Glycosyltransferase enables the use of glucose for the formation of extracellular polysaccharides, and increases bacterial adhesion (18). Vasquez et al. (2010) and Ribeiro et al. (2012) used S. mutans biofilm model for the assessment of the efficacy of antimicrobial agents and enamel demineralization. They showed that 0.05% NaF, unlike 0.12% chlorhexidine, had no significant effect on biofilm formation, or S. mutans colony count; however, it decreased enamel demineralization. Thus, the physio-mechanical effects of 0.05% NaF mouthwash in the process of progression of carious lesions are attributed to decreasing demineralization and increasing remineralization, and not its antibacterial activity (19, 20). Their results were in agreement with our findings. Jothika et al. (2015) (20) and Sadat Sajadi et al. (2015) (21) evaluated the effect of 0.2% NaF mouthwash on oral S. mutans in a clinical trial. They found that NaF mouthwash significantly decreased S. mutans plaque compared with the control group. Difference between their results and ours may be attributed to unequal amount of fluoride ions in mouthwashes because the antibacterial effects of fluoride are dose-dependent. 

The results of the present study indicated that 0.005% GO, and 0.005% GO-0.05% NaF mouthwashes effectively decreased S. mutans count in the saliva of rats compared with the control group. Furthermore, these two mouthwashes were more effective than 0.05% NaF alone. The most acceptable mechanism for antibacterial activity of graphene is via physical impairment of the cell membrane, oxidative stress, and entrapment or wrapping (22, 23). Moreover, GO impairs the integrity of cell membrane and cell wall (2). It is assumed that modified functional groups on GNPs play an important role in mediating oxidative stress. Nonetheless, due to physical and chemical complexities of GO, the precise correlation of functional groups with antibacterial activity has not been well elucidated (23). 

In line with our findings, some other studies also reported the antibacterial effects of GO. Zhao et al. (2020) (23) evaluated the antibacterial effects of GO sheets (40 µg/mL) containing functional groups on S. mutans. They found that the effects of GO sheet on S. mutans biofilm and S. mutans in planktonic form were dose-dependent. Besides, GO functional groups played a critical role in antibacterial activity. He et al. (2015) (2) reported that GO nano-sheets decreased the viability and number of S. mutans in vitro.

In the present study, maximum percentage of inflammation was noted in NaF-GO (94.4%) followed by GO (83.3%) and NaF (27.8%) groups. Type of inflammation was chronic, and the severity of inflammation was mild in 83.3% of the cases in NaF-GO group, 66.73% of the cases in GO group, and 27.8% of the cases in NaF group. Either ulceration or severe inflammation was not seen in any group. Gabler showed that fluoride ions can be absorbed through the oral mucosaof rats (24) . 

Incubation of oral mucosal fibroblasts with NaF at concentrations of 4 mmol/L (80 PPM) or higher within 24 h elicited a cytotoxic response which was concentration- and time-dependent (25). In this study, chronic mild inflammation observed in rats in 0.05% NaF group can be due to the long-term exposure of buccal mucosa and hard-to-reach areas (due to inadequate irrigation) to fluoride, and residual fluoride remaining in some parts of the oral cavity. 

Regarding the inflammatory effects of GO, Rodrigues et al. (26) exposed the lungs to functionalized GO (50 µg per mouse) and showed that lateral dimensions played an important role in pulmonary response to GO in short-term and long-term after single exposure in mice. Histological assessment of the lungs showed acute inflammatory response at 1 and 7 days after instillation. Micrometer-sized GO generated the most severe adverse reaction including chronic inflammation and formation of non-necrotizing peri-bronchiolar granulomas that lasted for 90 days. However, the mice exposed to nano-meter sized GO showed complete histological recovery by day 28. To date, no study has evaluated the histopathological effects of GO on the oral mucosa. In the present study, GO nanoparticles measuring 3.4 to 7 nm in size were used. It appears that very small GO particles are highly flexible and easily pass through the oral mucosal membrane. Thus, if they are not adequately washed away after use in the form of mouthwash, they can cause mucosal inflammation due to long-term exposure; although the chronic inflammation developed following the use of GO mouthwash was mild in the majority of cases in the present study. 

**In vitro study results (enamel microhardness): **Hardness is an important mechanical property of materials, which refers to resistance of a material or surface against indentation or penetration. Since the superficial layer of the enamel plays a fundamental role in progression of dental caries (18), assessment of changes in this layer is highly important. In the present study, microhardness was measured by the Vickers hardness tester (27). This technique is simple and highly precise, enables quantitative measurements, and is reproducible (27, 28). 

Evidence shows that NaF in different concentrations and forms such as mouthwash and varnish, can significantly enhance remineralization (29). In the present study, application of 0.05% NaF mouthwash enhanced remineralization without affecting the S. mutans count. Application of 0.005% GO mouthwash in our animal study significantly decreased S. mutans count in the saliva of rats. Moreover, our in vitro study showed that it did not significantly change the enamel microhardness in the presence of S. mutans due to its high antimicrobial activity and subsequent prevention of demineralization. However, 0.05% NaF-0.005% GO mouthwash significantly decreased the S. mutans count and increased enamel microhardness. Thus, further studies are required on the combination of 0.05% NaF and 0.005% GO mouthwash in patients at high risk of caries with high level of S. mutans because a combination of these two mouthwashes has two optimal properties namely high antibacterial activity due to the presence of GO and optimal remineralizing effect due to the presence of fluoride. One limitation of this study was the fact that the role of saliva in diluting the 0.005% GO mouthwash and decreasing its antibacterial effect was disregarded (since many in vitro studies have shown that GO has a cytotoxicity threshold for mammal cells). Future studies on higher concentrations of GO are warranted. 

 In conclusion, the addition of GO nanoparticles improved the antibacterial properties without causing adverse mucosal effects such as ulceration, acute inflammation or atrophy of the epithelium in the oral mucosa, but had no effect on the surface hardness of the enamel.
